# Down-regulation of ACACA suppresses the malignant progression of Prostate Cancer through inhibiting mitochondrial potential

**DOI:** 10.7150/jca.49560

**Published:** 2021-01-01

**Authors:** Hui Zhang, Shaoyou Liu, Zhouda Cai, Weimin Dong, Jianheng Ye, Zhiduan Cai, Zhaodong Han, Yuxiang Liang, Yangjia Zhuo, Yong Luo, Xuejin Zhu, Yulin Deng, Yixun Zhang, Ren Liu, Yuanfa Feng, Jiarun Lai, Rui Zhou, Huijing Tan, Weide Zhong

**Affiliations:** 1 Department of Urology, Guangzhou First People's Hospital, School of Medicine, South China University of Technology, Guangzhou, Guangdong, 510180, China.; 2 Guangdong Provincial Institute of Nephrology, Nanfang Hospital, Southern Medical University, Guangzhou, Guangdong, 510515, China.; 3 Urology Key Laboratory of Guangdong Province, The First Affiliated Hospital of Guangzhou Medical University, Guangzhou Medical University, Guangzhou, Guangdong, 510230, China.; 4 Department of Urology, Huadu District People's Hospital, Southern Medical University, Guangzhou, Guangdong, 510800, China.

**Keywords:** ACACA, Mitochondria, DU145, PC3, Prostate cancer

## Abstract

**Background and aim:** Silencing the expression of ACACA inhibits cell proliferation and induces apoptosis in prostate cancer LNCaP cells. However, the role of ACACA in other prostate cancer cells is not fully understood. Also, the effect of knocking down ACACA gene on mitochondria remains unclear. This study aimed to discover the specific role of ACACA gene in prostate cancer (PCa) DU145 and PC3 cells as well as its effects on mitochondrial potential.

**Methods:** The expression of ACACA gene was detected in human prostate cancer tissue microarrays and assessed in different clinical stages. Then, prostate cancer cell lines with low expression of ACACA were constructed to evaluate the changes in their cell cycle, proliferation, and metabolites. The effect of ACACA on tumor formation *in vivo* was analyzed. Also, mito-ATP production, mitochondrial staining, and mtDNA, nicotinamide adenine dinucleotide (NAD+/NADH), and reactive oxygen species (ROS) levels were detected.

**Results:** ACACA was expressed more strongly in prostate cancer tissues. The expression level of ACACA was higher in patients with advanced PCa than in patients with lower grades. The proliferation ability reduced in ACACA-knockdown cells. In* in vivo* tests, the tumor volume and weight were lower in the experimental group than in the control group. Mito-ATP production decreased significantly after ACACA suppression, mtDNA levels and MitoTracker staining decreased in the experimental group. The ratio of NAD+/NADH and ROS levels were upregulated in the experimental group.

**Conclusion:** Targeting ACACA gene and mitochondria might serve as a novel therapy for prostate cancer treatment.

## Introduction

Studies have shown that many malignant tumors prefer aerobic glycolysis 1,2. However, abnormal activation of certain oncogenes or inactivation of specific tumor suppressor genes can lead to the development of specific cancers 3,4,5. Extensive evidence shows that the overexpression of certain lipogenic enzymes 6, especially fatty acid synthase (FAS) 7,8, is a common feature in tumor cells, including in gastric cancer, lung cancer, breast cancer 8,9,10 and prostate cancer 11,12,13. ACACA, which is the rate-limiting enzyme of FAS, works as a catalyst for the carboxylation of CO_2_ and conversion of acetyl-CoA into malonyl-CoA. ACACA is highly enriched in adipogenic tissues. The enzyme is under long-term control at the transcriptional and translational levels through targeted phosphorylation/dephosphorylation of serine residues and allosteric transformation of citric acid or palmitoyl-CoA. An increasing number of recent studies focused on the role of ACACA in tumors. Raimondo et al. identified ACACA as an antitumor target of citrus lemon-derived nanovesicles in colon cancer 14. Ghoneum et al. showed that ovarian cancer stem cells also exhibited a significant increase in the expression of ACACA 15. ACACA is also essential for breast cancer cell survival 16. In addition, Garcia et al. showed that ACACA was the confluence point of EMT and promotion of invasion pathways in mouse and human breast tumors 17. Cetuximab-mediated activation of Adenosine 5'-monophosphate (AMP)-activated protein kinase and subsequent phosphorylation and inhibition of acetyl-CoA carboxylase were followed by a compensatory increase in the total acetyl-CoA carboxylase level, which rewired cancer metabolism from glycolysis-dependent to lipogenesis-dependent in head and neck squamous cell carcinoma 18. ACACA was overexpressed in liver cancer and contributed to the proliferation of human hepatoma G2 cells and the rat liver cell line BRL3A 19. Besides, it promoted glucose-mediated fatty acid synthesis, thus improving the survival rate of mice and patients with hepatocellular carcinoma 20. ACACA decreased the synthesis of fatty acids and could be used as a therapeutic target in metabolic syndrome 21. Studies have shown that silencing the ACACA gene may result in the inhibition of cell proliferation and induction of apoptosis in highly lipogenic prostate cancer LNCaP cells 22. However, the role of ACACA in non-androgen-dependent prostate cancer DU145 and PC3 cells and the response of mitochondria to ACACA gene suppression in the two cell lines remain unclear.

This study tested the effects of down-regulation of ACACA in non-androgen-dependent prostate cancer (PCa) cells and detected its effects on mitochondria. The data showed that the expression level of ACACA was higher in patients with prostate cancer than in healthy controls, which was consistent with the Gene Expression Profiling Interactive Analysis (GEPIA) data. The expression of ACACA was higher in patients with advanced PCa than in patients with lower grades. The proliferation ability reduced in ACACA-knockdown cells (experimental group). The *in vivo* study showed that tumor growth was inhibited in the experimental group. In this study, mitochondrial potential refers to the mitochondrial function and mitochondria bioenergetic profile. Mitochondrial function could be tested through the Seahorse XF96 Cell Mito Stress Test, mitochondria bioenergetic profile could be tested through the qRT-PCR of mitochondrial DNA (mtDNA) and the MitoTracker staining. Data indicated that mitochondrial potential also decreased in the experimental group. Additionally, the balance of nicotinamide adenine dinucleotide (NAD+/NADH) was disrupted, and the production of reactive oxygen species (ROS) was upregulated in the experimental group. The results suggested that ACACA was vital in prostate cancer, and the down-regulation of ACACA suppressed prostate cancer through inhibiting mitochondrial potential. The findings provided scientific evidence for future research on prostate cancer.

## Materials and Methods

### Tissue microarrays

Tissue microarrays (TMAs) were purchased from Alenabio (Xi'An, China), containing 69 prostate cancer tissues and 7 normal prostate tissues for further immunohistochemical (IHC) staining. Patients with PCa were never treated. Tissue information is summarized in Supplementary Table 1. The immunohistochemically stained regions were classified as follows: 0, 0%; 1, 1%-25%; 2, 26%-50%; 3, 51%-75%; and 4, 76%-100%. The staining intensity was determined visually and defined as follows: 0, negative; 1, weak; 2, moderate; and 3, strong. The final immunoreactivity score in each case was calculated by multiplying the area percentage by intensity scores.

### Cell culture and transfection

Both human prostate cancer cell lines DU145 and PC3 were purchased from the American Type Culture Collection (VA, USA) and cultured in Dulbecco's modified Eagle's medium (Gibco, USA) supplemented with 10% fetal bovine serum (Gibco) and 5% antibiotics (penicillin-streptomycin, Gibco). The cells were incubated in a 5% CO_2_ incubator at 37°C. Two stable cell lines (DU145 and PC3 cells) with the down-regulation of ACACA gene were produced by HYY Med (Guangdong, China) using a lentivirus.

### Quantitative reverse transcription-polymerase chain reaction and Western blot analysis

Quantitative reverse transcription-polymerase chain reaction (qRT-PCR) and Western blot assay were performed according to the protocol described previously 23. The qRT-PCR primers were as follows: β-actin (forward primer: AGCGAGCATCCCCCAAAGTT, reverse primer: GGGCACGAAGGCTCATCATT); ACACA (forward primer: AGGAGCTGTCTATTCGGGGT, reverse primer: GGTCGCTCAGCCTGTACTTT); mtDNA (forward primer: CACTTTCCAGACATCA, reverse primer: TGGTTAGGCTGGTGTTAGGG). The primary antibodies used for Western blot analysis were ACACA (Boster, China) and β-actin (Boster, China).

### Cell cycle analysis

The cells were fixed with ethanol, centrifuged at 3000 rpm for 5min, and washed twice with phosphate buffer. Then, the cells were incubated with 0.5 mL of phosphoric acid containing 100 µg/mL RNase A, 5 µg/mL propidium iodide, and 0.3 µL of 0.3% Triton X-100 for 30 min at 4 °C protected from light. The cell cycle distribution was analyzed using a BD-FACS Calibur (BD Biosciences Pharmingen, USA).

### Cell counting kit 8

A total of 5000 cells were seeded into the 96-well plate and cultured in an incubator (37 °C, 5% CO_2_) for 4, 24, 48, and 72 h. Then, 20 µL of cell counting kit 8 (CCK-8) solution was added to each well and incubating for 2h. After that, the absorbance at 450 nm was measured with a microplate reader.

### Generation of an *in vivo* xenograft model

For *in vivo* tumor formation assays, DU145 cells were transfected with sh-ACACA-c or sh-Negative Control (sh-NC) lentiviral vectors. The cells were treated with trypsin, suspended in phosphate-buffered saline (PBS), and mixed with the matrix gel (catalog number 356234, BD Biosciences) at a ratio of 1:1. The DU145-sh- ACACA-c cell line was injected into the right side of each male nude mouse, while the DU145-sh-NC cell line was injected into the left of the same nude mouse (*n* = 11). The cell number was 2 × 10^6^. The tumor size was measured every 4 days, and the tumor volume was calculated: *V* (mm^3^) = 0.5 × width (mm) × length (mm)^2^. The mice were sacrificed by CO_2_ asphyxiation. The animal assays were performed in accordance with the principles and procedures of the “Guidelines for the Care and Use of Laboratory Animals”. The proposed experimental plan and procedures were reviewed and approved by the ethics committee of the First People's Hospital of Guangzhou, affiliated to the South China University of Technology. All experiments were conducted in strict accordance with relevant guidelines and regulations.

### Histology

Hematoxylin and eosin staining and IHC staining were performed as previously described 24 with the antibody of proliferating cell nuclear antigen (Anti-PCNA) (1:100, catalog number: M00125, Boster, China).

### Liquid chromatography-mass spectrometry (LC-MS)

The cells (1×10^7^) were prepared for the metabolite extraction. The extract containing internal standard (1000 µL) (methanol: acetonitrile: water volume ratio = 2:2:1, internal standard 2 µg/mL) was transferred to an Eppendorf tube (EP) in three separate steps and vortexed for 30 s. Porcelain beads were added, and then sonicated for 5 min (ice water bath). The aforementioned steps were repeated three times. The samples were incubated for 1h at -20 °C to precipitate proteins. The sample was centrifuged at 12,000 rpm for 15 min at 4 °C. The supernatant was transferred to the EP tube. The extract was dried in a vacuum concentrator. The dried metabolites were reconstituted by adding 200 µL of the extract (acetonitrile: water volume ratio = 1:1). The samples were vortexed for 30 s and sonicated for 10 min. They were centrifuged at 4 °C and 12,000 rpm for 15 min. The supernatant (75 µL) was transferred into a 2 mL injection vial. Then, 10 µL of each sample was considered as a quality control sample. Further, 75 µL of each sample was used for the LC-MS analysis.

### Seahorse assay

The oxygen consumption rate (OCR) and extracellular acidification rate (ECAR) were detected using an XF96 extracellular flux analyzer (Agilent, USA) to determine the Mito-ATP production. Briefly, 18,000 prostate cancer cells per well were seeded into specific culture plates in a complete medium overnight. The cells were washed and incubated with a basic medium containing 2 mM L-glutamine for 1 h at 37 °C and then incubated in a CO_2_-free incubator to guarantee precise measurements of extracellular pH. OCR and ECAR measurements were conducted following the manufacturer's protocols. For the Mito Stress Test (Kit 103015-100), data were analyzed under basal conditions or in response to 1μM oligomycin (OLI), 1 µM Fluorocarbonyl-cyanide-phenylhydrazone (FCCP), and 0.5 µM rotenone/antimycin A (Rtn/AA). For the real-time ATP rate assay (Kit 103592-100), the concentration of the drug was changed to 1.5 µM and 0.5 µM for OLI and Rtn/AA, respectively. For the glycolytic rate assay (Kit 103344-100), the cells were detected after adding 0.5 µM Rtn/AA and 50 mM 2-deoxy-D-glucose (2-DG) (seahorse kits all from Agilent, USA).

### MitoTracker test

When the cells reached the desired density (0.7×10^6^/well) in a six-well plate, the media was discarded, and a prewarmed staining solution containing a MitoTracker probe was added (Invitrogen, USA; work solution 80 nM). The cells were incubated for 30 min in a humidified incubator at 37 °C with 5% CO_2_. They were washed with PBS twice and trypsinized into a single cell suspension. They were resuspended in 300 µL of PBS for each tube, transferred to a flow tube, and analyzed by flow cytometry. After staining was completed, the staining solution was replaced, 4% paraformaldehyde was added to the solution and fixed for 20 min, and 1% TritonX-100 was added to the solution and incubated for 10 min. Further, 0.5% diamidino-2-phenylindole (DAPI) was added to the solution and then incubated for 7min. PBS was used to wash the cells three times. The cells were observed under a fluorescence microscope (Lecia DNMIL LED, China).

### Detection of NAD+/NADH

A total of 2×10^6^ cells were obtained, the culture solution discarded, and 200 µL of NAD+/NADH extract buffer was added and gently pipetted to promote cell lysis. This was followed by centrifugation at 12,000 *g* and 4°C for 10min and testing of the supernatant. The NADH standard and alcohol dehydrogenase working solutions were prepared according to the instruction manual. Next, 100 µL of the sample was tested in a centrifuge tube and heated on a water bath (60 °C) to decompose NAD+. Blank control wells, standard wells, or sample wells were set up using a 96-well plate. The alcohol dehydrogenase working solution was added and mixed gently. The NAD+/NADH ratio was determined according to the instruction manual (NAD+/NADH colorimetric detection kit: Beyotime, China).

### Detection of ROS levels

The prepared cell suspension was transplanted into a 6-well plate (0.6×10^6^ /well) and placed in a conventional incubator for 24 h. The complete medium was discarded, and 1000 µL of fresh serum-free medium was added to each well. Further, 1 µL of dihydroethidium (DHE, BestBio, China) was added to each well, gently mixed, and placed in the incubator for 30 min. The cells were washed with PBS twice and trypsinized into a single cell suspension. They were resuspended in 300 µL of PBS in each tube, transplanted into a flow tube, and analyzed by flow cytometry.

### Statistical analysis

All assays were performed for more than three independent experiments. Data were reported as mean ± standard deviation. Data were analyzed using SPSS 20.0 and Prism 7.0. According to data distribution, a two-tailed unpaired-sample Student *t* test was used to compare the mean values between different groups (^*^P < 0.05, ^**^P < 0.01, ^***^P < 0.001 compared with the control group).

## Results

### ACACA was upregulated in prostate cancer tissues, and the expression was related to the TNM stage

**Figure 1:**
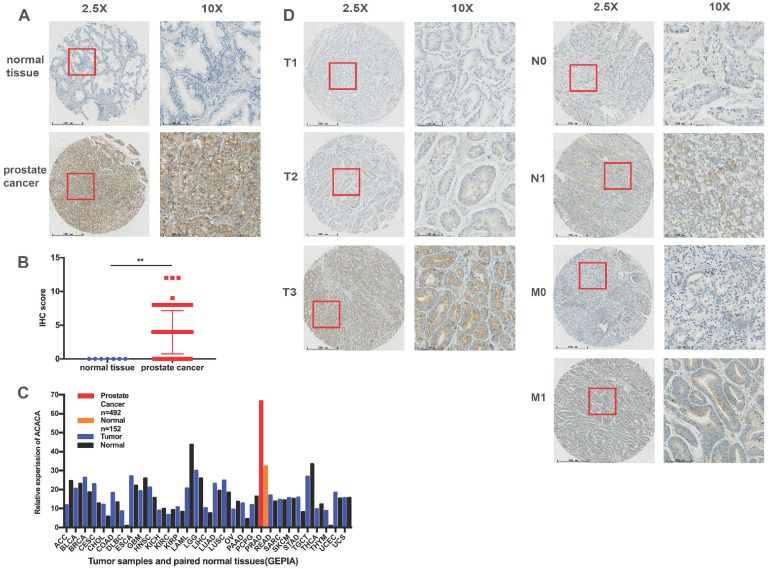
** ACACA was upregulated in prostate cancer tissues, and the expression was related to the TNM stage.** (**A**) Immunohistochemistry staining images of ACACA protein in normal tissue and prostate cancer tissue under different views. (**B**) Immunohistochemistry revealed ACACA protein expression score higher in prostate cancer than the normal tissue (prostate cancer *n* =69, normal tissue *n* =7). (**C**) ACACA mRNA expression level in 31 tumors and their paired normal tissues from GEPIA public database were analyzed. The full name of 31 tumor abbreviations were shown in Supplementary Information. (**D**) The representative images of ACACA staining in different stages of prostate cancer patients were presented (T: Primary tumor, N: Regional lymph node, M: Distant metastasis; 2.5X, magnification, scale bar: 500 um; 10X, magnification; scale bar: 100 um). Data were presented as Mean ± SD. **P < 0.01.

A TMA comprising 69 PCa tissues and 7 normal tissues was employed to analyze the protein expression of ACACA. Immunohistochemical staining showed that ACACA was mainly expressed in the cytoplasm of prostate cancer cells (Figure 1A and 1D). However, the expression of ACACA was not found in the normal tissues because of the limited number of cases (Figure 1A-B). To verify the results, first the mRNA levels of ACACA were analyzed in 31 tumors on the public database GEPIA (25). The expression of ACACA was significantly higher in prostate cancer (*n* = 492) than in the normal tissues (*n* = 152) (Figure 1C). Then, the expression of ACACA in the PCa tissues was analyzed at different clinical stages. The image revealed that the expression of ACACA was significantly upregulated in T3, N1, and M1 compared with T1-T2, N0, and M0, respectively (Figure 1D). In a word, the expression level of ACACA was higher in patients with advanced PCa than in patients with lower grades (Table 1). Detailed immunohistochemical staining scores of ACACA in tissues are shown in Supplementary Table 1.

### Proliferation ability and tumor formation decreased in prostate cancer cells after knocking down ACACA gene *in vitro* or *in vivo*

**Figure 2:**
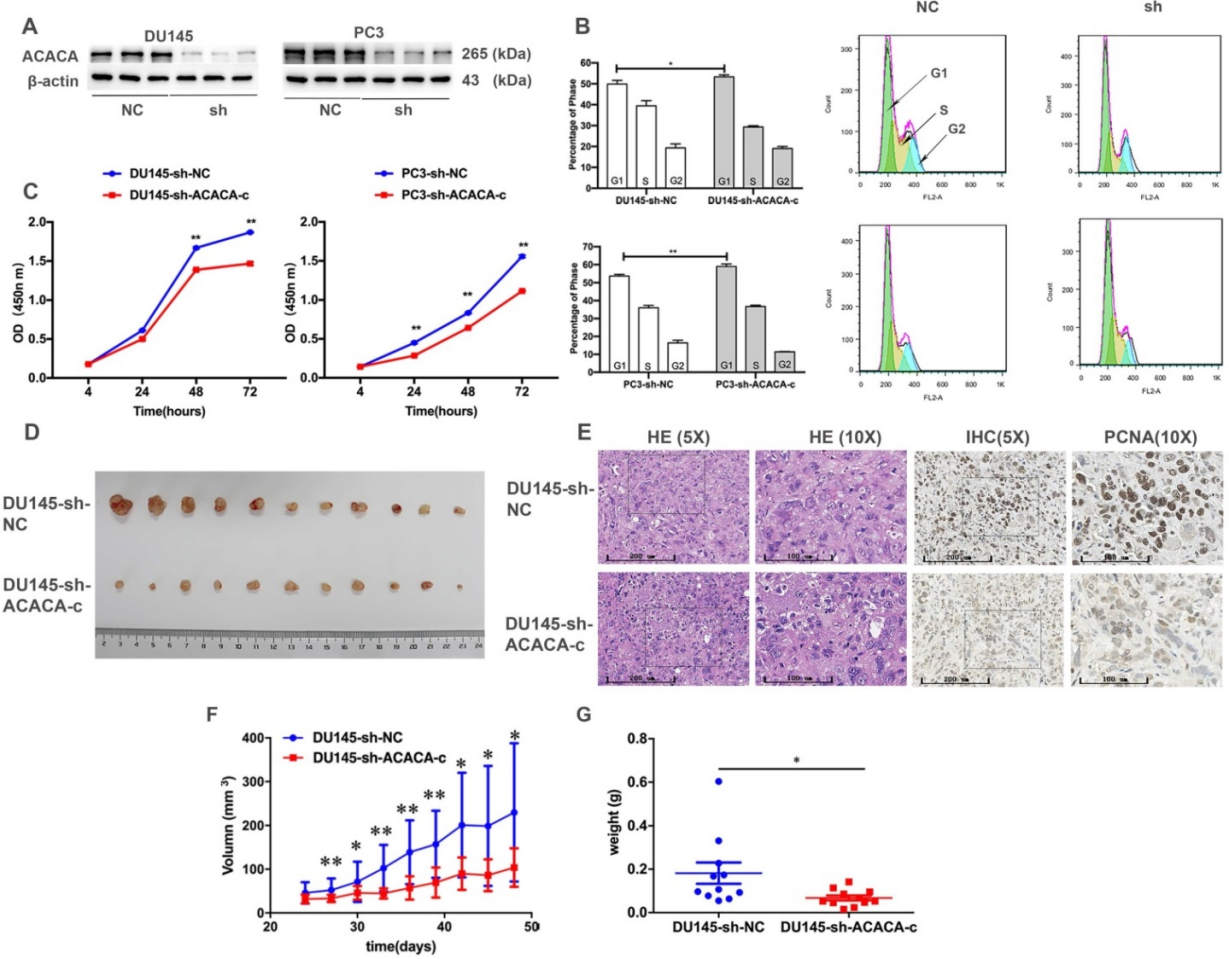
** Proliferation ability and tumor formation decreased in prostate cancer cells after knocking down ACACA gene* in vitro* or *in vivo*.** (**A**) Western blot detected the effect of the sh-ACACA-c vector transfected in DU145 and PC3 cells. (**B**) Cytometry flow showed that ACACA knockdown resulted in an extension of G1 phase of cell cycle in DU145 and PC3 cells. (**C**) CCK-8 assay showed the ability of proliferation in prostate cancer cells after knocking down the ACACA gene. (**D**) Inhibition of ACACA gene in DU145 cells significantly retarded subcutaneous tumor growth in nude mice (*n*=11). (**E**) Hematoxylin and eosin (H&E) and Immunohistochemistry (IHC) staining of tumor tissue. (**F-G**) The tumor volume curve and weight were shown. Data were presented as Mean ± SD. *P < 0.05. **P < 0.01.

The cell cycle and CCK-8 assays were performed to evaluate the influence of ACACA on the proliferation of PCa cells. First, two cell lines having the lower expression of ACACA gene were constructed (DU145-sh-ACACA-c, PC3-sh-ACACA-c, and relevant control cell lines). The protein expression level of ACACA in the cell lines is shown in Figure 2A. The cell lines were successfully constructed, and the protein levels of ACACA were significantly downregulated in the two cell lines. The cell cycle was also extended in the G1 phase in the experimental group (DU145-sh-ACACA-c or PC3-sh-ACACA-c) compared with the negative control group (DU145-sh-NC or PC3-sh-NC) (Figure 2B). The CCK-8 assay indicated that down-regulation of ACACA significantly reduced cell viability (Figure 2C). The DU145-sh-NC and DU145-sh-ACACA-c cell lines were used to detect the effect of ACACA on the tumor growth in nude mice so as to test the *in vivo* biological function of ACACA. DU145-sh-ACACA-c cells were injected subcutaneously into the right side of each male nude mouse, while the DU145-sh-NC cells were injected into the left side of the same nude mouse. DU145-sh-ACACA-c cells formed significantly smaller tumor nodules (Figure 2D). Despite no significant differences in tissue histology among the DU145-sh-NC and DU145-sh-ACACA-c xenografts, the staining of PCNA was weaker than that in the control group, indicating that the knockdown of ACACA decreased cell proliferation in the xenografts (Figure 2E). Figure 2F shows that the tumor volume was smaller in the DU145-sh-ACACA-c group than in the DU145-sh-NC group after 27, 33, 36, and 39 days (*P* <0.01). The tumor volume was smaller in the DU145-sh-ACACA-c group than in the DU145-sh-NC group after 30, 42, 45, and 48 days (*P* < 0.05). Figure 2G shows that the tumor weight was lower in the DU145-sh-ACACA-c group than in the DU145-sh-NC group (*P* < 0.05). These results strongly proved that ACACA significantly affected tumor growth *in vivo*.

### ATP production in DU145 and PC3 cell lines reduced after knocking down ACACA gene

**Figure 3:**
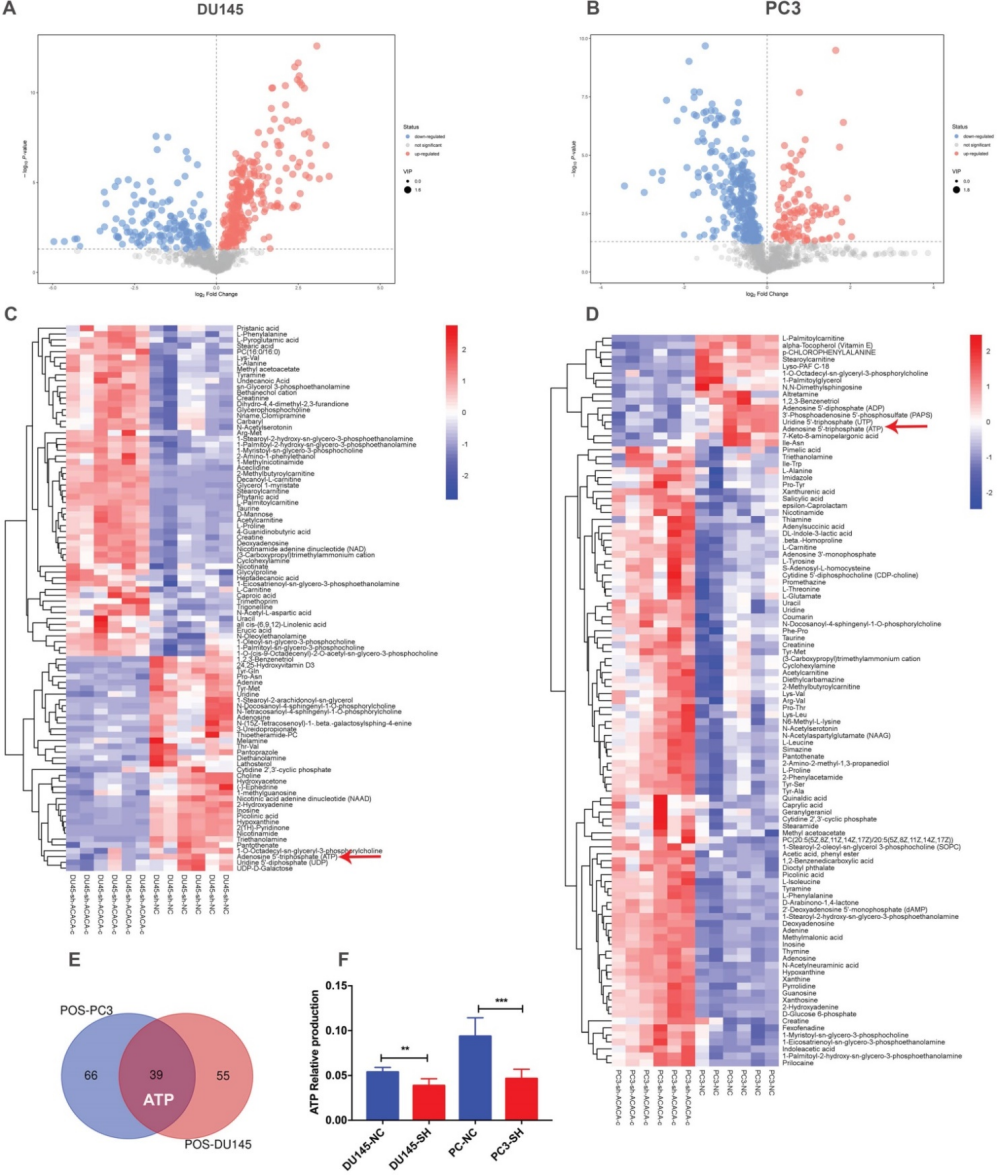
** ATP production in DU145 and PC3 cell lines reduced after knocking down ACACA gene.** (**A-B**) Volcano plot of differential metabolites in the four cell lines. (**C-D**) Heatmap analysis of the differential metabolites in the prostate cancer cells. Relative metabolite levels were described in terms of color gradations. Red indicated up, blue indicated down. Red arrow indicated the relative production of ATP. (**E**) Overlap of metabolites that have changed in the cell lines. (**F**) Quantitative analysis of ATP production in metabolomics. Data were presented as Mean ± SD. **P < 0.01. ***P < 0.001.

Since ACACA is an enzyme involved in fatty acid metabolism, the changes in metabolomics after knocking down ACACA gene were detected by nontargeted metabolomics. All the cell samples in the score map were within the 95% of Hotelling's T-squared ellipse, implying no outlier among the analyzed samples. Then, the different metabolic patterns were explained. These data suggested clear separation and differences between the two groups. The volcano plot showed the changes in metabolites in the experimental group (Figure 3A-B). In the LC-MS assay, 94 metabolite features in DU145-sh-ACACA-c and 105 metabolite features in PC3-sh-ACACA-c were identified. The metabolite change trend was visually divided into upregulated and downregulated groups. Among the 94 identified metabolites, 37 were downregulated while 57 were upregulated in DU145-sh-ACACA-c cells. In PC3-sh-ACACA-c cells, 16 were downregulated while 89 were upregulated (Figure 3C-D). Among these, 39 metabolites, including lipids, amino acids, carboxylic acids, and their derivatives, changed in the experiment group (Supplementary Table S2 and Table S3). The 39 substances were included in the enrichment analysis, revealing that substances were enriched mainly in the beta-oxidation of very long-chain fatty acids and purine metabolism pathway (Supplementary Figure 1). The main hits in the beta-oxidation pathway of very long-chain fatty acids were L-carnitine, acetylcarnitine, and ATP. L-carnitine and acetyl-carnitine were the substrates of fatty acid beta-oxidation. Further, fatty acid beta-oxidation produced ATP, which provided energy for tumor cell proliferation. In the purine metabolism pathway, adenine, adenosine, deoxyadenosine, hypoxanthine, inosine, and ATP were the main hits. ATP production changed in the two metabolism pathways. Besides, among the common metabolites, ATP was downregulated in both experimental group (Figure 3C-F) with statistically significant differences. Data indicated that down-regulation of ACACA gene affected mainly the beta-oxidation of very long-chain fatty acids and purine metabolism pathways. The specific mechanism needs to further exploration.

### Mitochondrial function was reduced on knocking down ACACA gene in DU145 and PC3 cell lines

**Figure 4:**
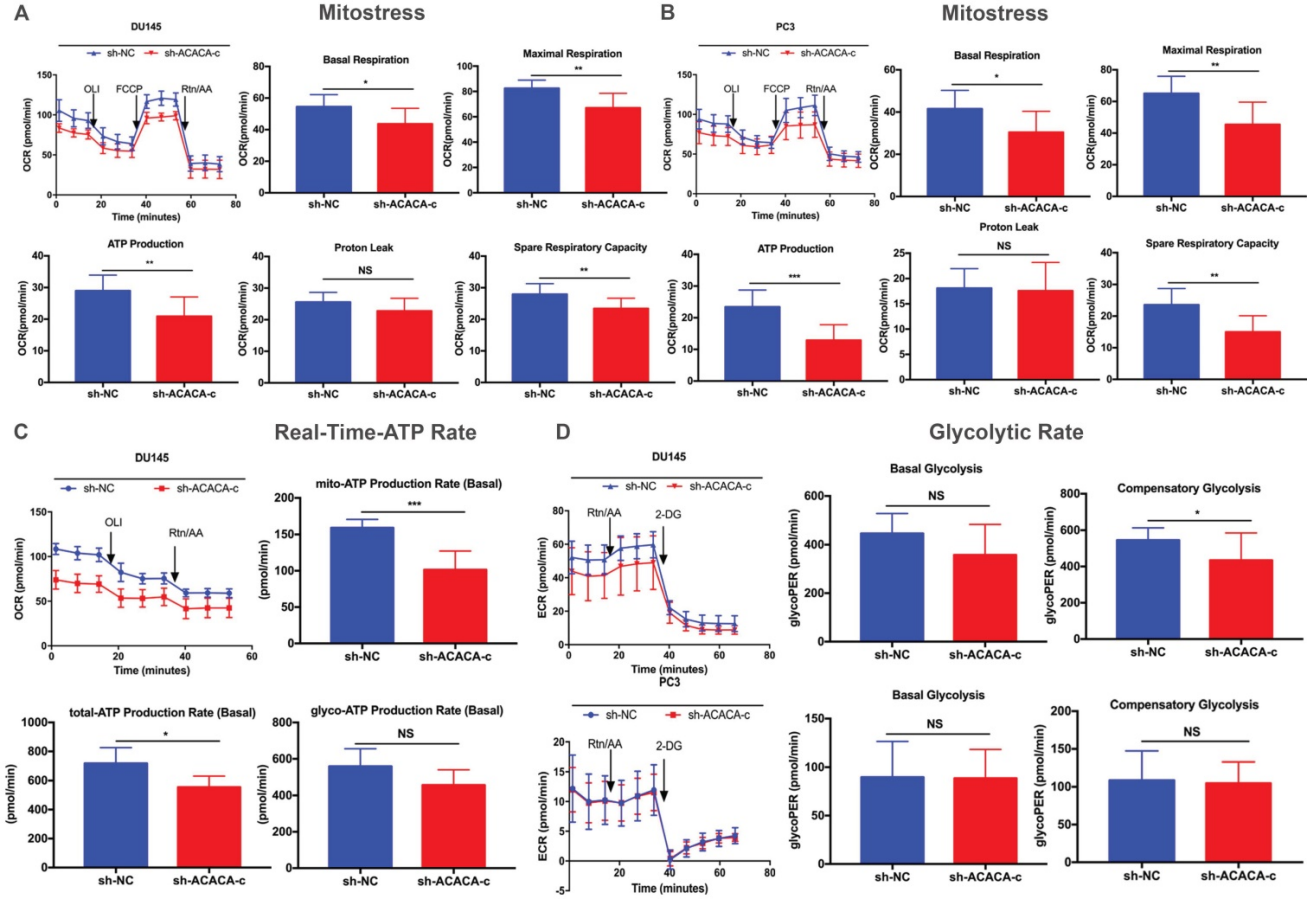
** Mitochondrial function was reduced on knocking down ACACA gene in DU145 and PC3 cell lines.** (**A-B**) Detected OCRs in the DU145 and PC3 cells by seahorse XF96 Mito-Stress kit under basal conditions and in response to specific inhibitors. The fundamental parameters of mitochondria function were presented through a histogram. (**C**) OCR in the DU145 cells was analyzed by the seahorse XF96 Real-Time-ATP-Rate kit. ATP production was analyzed in the bar graph. (**D**) ECR indicated the glycolytic rate in DU145 and PC3 cells by the seahorse XF96 Glycolytic-Rate kit. Data were presented as Mean ± SD. *P < 0.05. **P < 0.01. *** P < 0.001.

ATP production was downregulated in the ACACA-knockdown cells. The study next detected the mitochondrial function in the four cell lines with a seahorse XF96 extracellular flux analyzer. Mitochondrial function refers to the OCR of cells, that is, the ability to consume oxygen to produce ATP, which could be tested using the Seahorse XF96 Cell Mito Stress Test. In the Mito Stress Test, the basal respiration, maximal respiration, and spare respiratory capacity were all downregulated, except proton leak, in the experimental group, suggesting a reduction in mitochondrial function in the experimental group (Figure 4A- B). Additionally, both experimental cell lines showed lower mito-ATP production (Figure 4A -B). The study further detected the whole ATP production in DU145 cell lines. The total ATP production and the level of mito-ATP decreased in the experimental group, consistent with previous findings. However, the glyco-ATP production did not show any difference in the cell line (Figure 4C). Similar results were found in PC3 cells (Supplementary Figure 2). In the glycolytic rate assay, data suggested no effect of knocking down ACACA gene in DU145 or PC3 cells, which were consistent with previous findings (Figure 4D).

### ACACA affected the mitochondria bioenergetic profile

**Figure 5:**
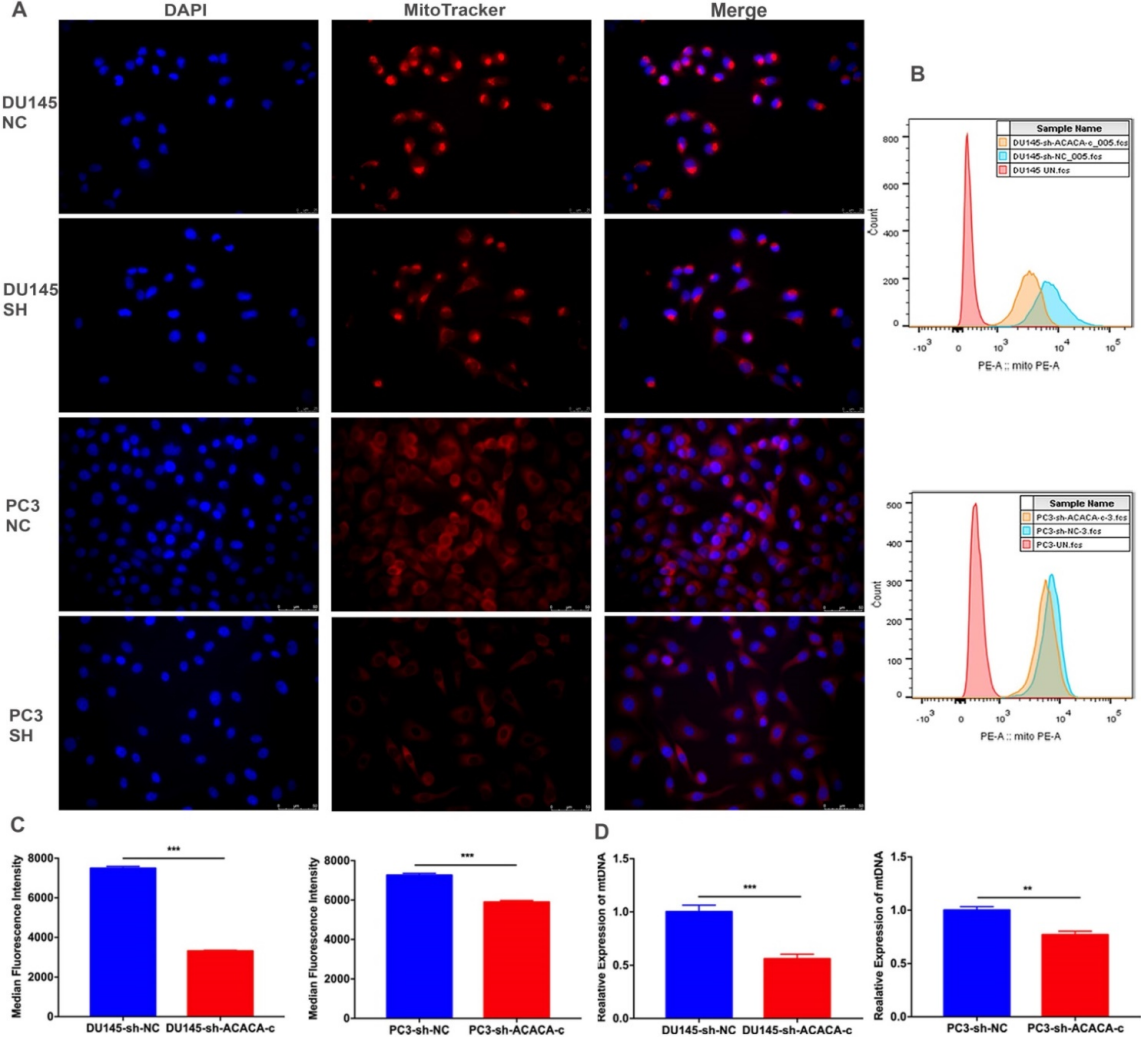
** ACACA affected the mitochondria bioenergetic profile.** (**A**) Fluorescence microscopy showed the staining of mitochondria (magnification was 40X). The nuclear of cells was stained by DAPI. Mitochondria was stained by the MitoTracker probe. (**B-C**) Flow cytometry was used to detect the MFI of mitochondria in the different cells. (**D**) qRT-PCR detected the mtDNA copy number of mitochondria. Data were presented as Mean ± SD. **P < 0.01. ***P < 0.001.

The mitochondrial function decreased on knocking down ACACA gene. The production of mito-ATP needs the participation of the oxidative phosphorylation system (OXPHOS). However, mtDNA, which encodes 13 polypeptides, is essential to OXPHOS. Also, mutations in mtDNA lead to a variety of diseases 26, 27. Some studies found that mitochondria could be a target for cancer 28. The present study detected the bioenergetic profile of mitochondria by detecting the MitoTracker and mtDNA in different cell lines. The results showed that the intensity of mitochondria and the median fluorescence intensity (MFI) of mitochondria were weaker in the experimental group than in the control group (Figure 5A-C). The mtDNA level also decreased in the experimental group, especially in the DU145-sh-ACACA-c cell lines (Figure 5D). The result suggested that ACACA influenced the activity of mitochondria, leading to the death of prostate cancer cells via affecting the ability to produce ATP and the mtDNA copy number. In summary, dysfunctional mitochondria were much more evident in ACACA-knockdown cells.

### Down-regulation of ACACA affected the NAD+/NADH and ROS level in DU145 and PC3 cells

**Figure 6:**
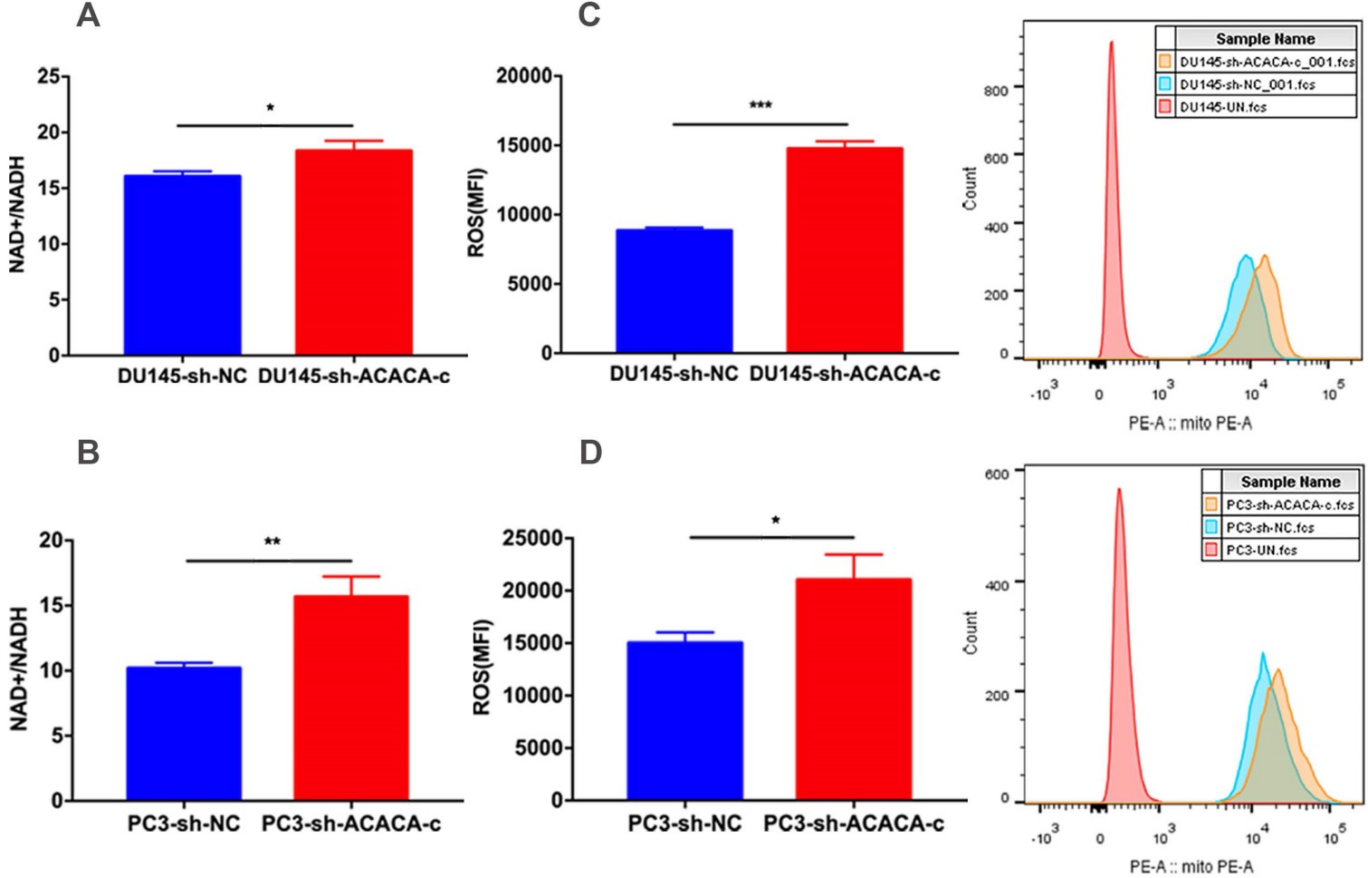
** Down-regulation of ACACA affected the NAD+/NADH ratio and ROS level in DU145 and PC3 cells.** (**A-B**) The NAD+/NADH colorimetric detection kit was used to analyze the NAD+/NADH ratio in DU145 cells and PC3 cells. (**C-D**) ROS levels were determined by staining with the DHE probe and observed through flow cytometry. The MFI was quantified on the left. Data were presented as Mean ± SD. *P < 0.05. **P < 0.01. ***P < 0.001.

The *in vitro* study showed that ACACA affected the proliferation ability in DU145 and PC3 cells and decreased the production of mito-ATP in the experimental group. The results of the MitoTracker assay and mtDNA suggested that the function of mitochondria changed in the experimental group. NAD+ is an essential co-factor in the production of ATP via glycolysis and OXPHOS. Studies have shown that the mitochondria complex I activity and the NAD+/NADH balance can regulate breast cancer progression. In addition, adjusting the ratio of NAD+/NADH inhibits tumor metastasis and prevents disease progression (29). The present study further detected the NAD+/NADH ratio. The NAD+/NADH ratio increased in the experimental cell lines as expected (Figure 6A-B). ROS production by mitochondria as a by-product of the electron transport chain can activate signal transduction pathways. Some studies have shown that the generation of ROS is crucial in the death of prostate cancer cells (30,31,32). Excessive ROS production may also lead to cell death. Further, the intracellular ROS levels were measured in the four cell lines. The data indicated an increase in ROS levels in experimental group (Figure 6C-D). The two results explained why the production of mito-ATP decreased in the DU145-sh-ACACA-c or PC3-sh-ACACA-c cells.

## Discussion

A recent study in the United States has shown that prostate cancer is the second cause of cancer-related mortality in human beings 33. Its incidence and mortality continue to increase due to the lack of specific biomarkers in the earlier stage of prostate cancer. Hence, new biomarkers need to be developed for the diagnosis of prostate cancer in the early stage. Many studies have indicated that ACACA is a potential target for cancer treatment 15-21. Brusselmans et al. found that the knockdown of ACACA resulted in a decrease in FAS, inhibition of proliferation, and induction of apoptosis in LNCaP prostate cancer cells. At the same time, no cytotoxic effect was observed 22. However, the aforementioned study was only an *in vitro* study in a single cell line; the effect of ACACA on mitochondria, which is an important site for energy production, was still unknown. Therefore, the present study focused on the other two prostate cancer cell lines DU145 and PC3 by observing their proliferation, cell cycle, cell metabolite changes, and mitochondrial characteristics after knocking down ACACA gene.

The expression of ACACA was first detected in a prostate cancer tissue chip. Its expression level in patients was significantly higher with prostate cancer. The same results were obtained from the GEPIA database. In addition, the expression of this gene was linked to the TNM stage. These findings suggested that ACACA might promote tumor growth in prostate cancer.

Next, two cell lines with low expression of ACACA were constructed, namely DU145-sh-ACACA-c and PC3-sh-ACACA-c cell lines and control cells. The cell cycle was blocked and the proliferation reduced *in vitro* after knocking down ACACA gene in prostate cancer. The results were the same as obtained in LNCaP cells 22. In addition, down-regulation of ACACA gene inhibited tumor formation in a nude mouse model.

The present study further discussed the metabolite changes in the four cell lines. It was noted that 94 substances in the DU45-sh-ACACA-c experimental group and 105 substances in the PC3-sh-ACACA-c experimental group changed. Further, 39 substances changed in both cell lines. Also, the production of ATP reduced at the same time, indicating that down-regulation of ACACA gene affected the energy metabolism of the two cell lines. Cancer cells produce energy by two mechanisms: extracellular glycolysis and oxidative phosphorylation in the mitochondria. The present study further explored the mechanism that was more important by detecting the production of ATP.

In the Mito Stress Test of both cell lines, the mitochondrial function, including basal respiration, maximal respiration, spare respiratory capacity, and mito-ATP, decreased after knocking down ACACA gene. The real-time ATP rate assay in DU45-sh-ACACA-c cells showed a decrease in total ATP and mito-ATP levels. The level of glyco-ATP decreased without any statistical significance, indicating that knocking down the gene decreased mito-ATP production without affecting glyco-ATP production. To confirm this hypothesis, glycolysis in the experimental group was analyzed by detecting the glycolytic rate. Down-regulation of the gene did not affect glycolysis. Overall, knocking down the gene affected mitochondria ATP production without glycolysis ATP production.

Down-regulation of ACACA gene affects mainly mitochondrial energy metabolism. In the MitoTracker assay by immunofluorescence or flow cytometry, the staining of mitochondria was weaker and the relative expression level of mtDNA was lower in the experimental group compared with the control group. The decreasing trend of mitochondrial staining was more palpable than PC3 cells because of different biological characteristics of the two cells, including metastatic potential and the expression of P53, E-cadherin, and Vimentin.

Mitochondrial function and mitochondria bioenergetic profile had changed in the experimental group. The mitochondrial potential changed, and the levels of NAD+/NADH and ROS were detected. Both NAD+/NADH and ROS levels increased in the experimental group. The NAD+ in mitochondria could not be transformed into NADH, and cell energy was insufficient probably due to impaired mitochondrial potential. It finally led to cell damage, and the intracellular ROS level increased. The present studies found that after knocking down the gene, 39 substances were changed, affecting mainly the long-chain fatty acid beta-oxidation and purine metabolism pathways. It was speculated that ACACA promoted the synthesis of fatty acids in the cytoplasm, leading to the entry of more substances into the mitochondria for beta-oxidation and more ATP production. At the same time, ACACA also promoted the synthesis of some genetic material (related to long-chain fatty acid beta-oxidation or cell membrane formation), ultimately promoting the growth of tumor cells. The specific mechanism needs further investigation.

## Conclusions

The high expression of ACACA gene has a positive correlation with the TNM stage of prostate cancer. The results confirmed that down-regulation of ACACA in prostate cancer cells affected the mitochondrial potential by mitigating the balance of NAD+/NADH, mitochondria ATP production, mtDNA, and ROS levels, which decreased the proliferation capacity of tumor cells. Testing mitochondrial potential and expression of ACACA might serve as a predictive target and a therapeutic method in the future.

## Supplementary Material

Supplementary figures and tables.

## Figures and Tables

**Table 1:** Association of ACACA expression score with clinicopathological parameters

Clinicopathological Parameters	Case no.	ACACA	
		Mean ± SD	*P*
**Age (years)**			
≥ 70	37	4.35 ± 3.075	0.274
< 70	32	3.50 ± 3.331	
**Regional lymph node (a)**			
N0	56	3.29 ± 2.762	**0.001**
N1	12	7.42 ± 2.906	
**Distant metastasis (a)**			
M0	59	3.47 ± 2.950	**0.001**
M1	9	7.56 ± 2.404	
**Primary tumor (a)**			
T1-T2	44	2.82 ± 2.213	**0.001**
T3-T4	24	6.21 ± 3.563	
**Pathological grade (b)**			
≥ 2	58	3.81 ± 3.154	0.897
< 2	5	4.00 ± 2.828	
**Tumor stage (a)**			
≥ 2	64	4.14 ± 3.206	0.195
< 2	4	2.00 ± 2.309	

a: one cancer samples could not be assessed; b: six cancer sample could not be assessed.
