# Supplementary material for: Down-regulation of ACACA suppresses the malignant progression of Prostate Cancer through inhibiting mitochondrial potential
Source: J Cancer. 2021 Jan 1;12(1):232–43. doi: 10.7150/jca.49560 (PMC7738814; doi:10.7150/jca.49560)
Supplement: Supplementary file 1 — Supplementary figures and tables. [file jcav12p0232s1.pdf]

Supplementary materials

(1) 31 tumor abbreviations

1. ACC Adrenocortical carcinoma
2. BLCA Bladder Urothelial Carcinoma
3. BRCA Breast invasive carcinoma
4. CESC Cervical squamous cell carcinoma and endocervical adenocarcinoma
5. CHOL Cholangiocarcinoma
6. COAD Colon adenocarcinoma
7. DLBC Lymphoid Neoplasm Diffuse Large B-cell Lymphoma
8. ESCA Esophageal carcinoma
9. GBM Glioblastoma multiforme
10. HNSC Head and Neck squamous cell carcinoma
11. KICH Kidney Chromophobe
12. KIRC Kidney renal clear cell carcinoma
13. KIRP Kidney renal papillary cell carcinoma
14. LAML Acute Myeloid Leukemia
15. LGG Brain Lower Grade Glioma
16. LIHC Liver hepatocellular carcinoma
17. LUAD Lung adenocarcinoma
18. LUSC Lung squamous cell carcinoma
19. OV Ovarian serous cystadenocarcinoma
20. PAAD Pancreatic adenocarcinoma
21. PCPG Pheochromocytoma and Paraganglioma
22. PRAD Prostate adenocarcinoma
23. READ Rectum adenocarcinoma
24. SARC Sarcoma
25. SKCM Skin Cutaneous Melanoma
26. STAD Stomach adenocarcinoma
27. TGCT Testicular Germ Cell Tumors
28. THCA Thyroid carcinoma
29. THYM Thymoma
30. UCEC Uterine Corpus Endometrial Carcinoma
31. UCS Uterine Carcinosarcoma

(2) Supplementary Figure 1

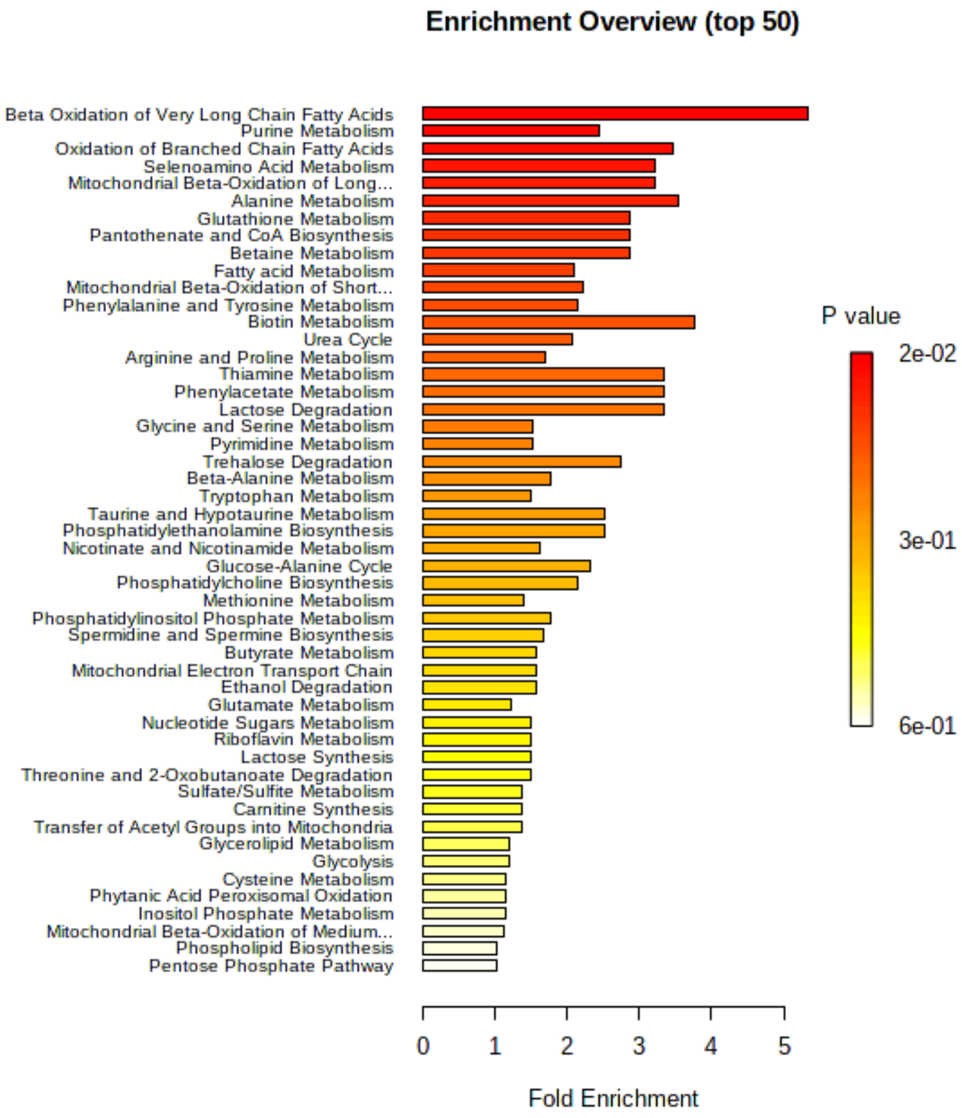

Summary Plot for metabolites analysis in DU145-SH and PC3-SH cell lines. Thirty-nine metabolites compounds which were both have changed including up-regulated and down-regulated in DU145-SH and PC3-SH cell lines were input for the Metabolite Set Enrichment Analysis.

(3) Supplementary Figure 2

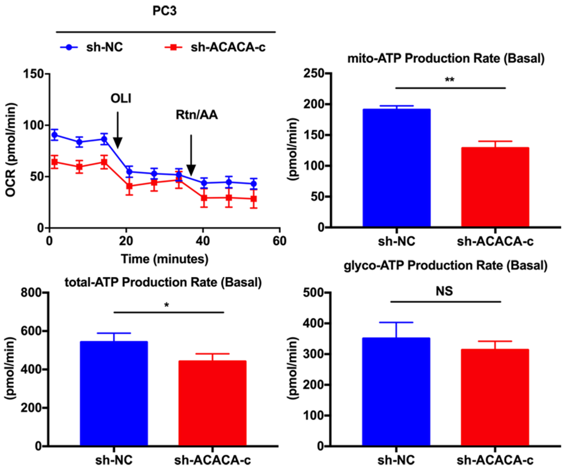

OCR in the PC3 cells was analyzed by the seahorse XF Real-Time-ATP-Rate kit. ATP production was analyzed in the bar graph.

(4) Supplementary Table 1

Position	No.		Age	Sex	Organ/Anator	Pathology diagnosis	n group	m group	t group	intensity	area	SCORE	TNM	Grade	Stage	Stage	Type	Tissue ID.
A1	1	1	71	M	Prostate	Adenocarcinoma	0	0	0	4	0	0	T2N0M0	2	II	2	Malignant	Mpr080049
A2	2	0	66	M	Prostate	Adenocarcinoma	1	1	1	4	2	8	T3N1M1	1	IV	4	Malignant	Mpr020491
A3	3	0	60	M	Prostate	Adenocarcinoma	0	0	1	4	0	0	T3AN0M0	3	III	3	Malignant	Mpr080010
A4	4	1	76	M	Prostate	Adenocarcinoma	0	0	0	4	0	0	T2AN0M0	1	II	2	Malignant	Mpr030277
A5	5	1	73	M	Prostate	Adenocarcinoma	0	0	0	4	2	8	T2N0M0	2	IIA	2	Malignant	Mpr130014
A6	6	0	26	M	Prostate	Adenocarcinoma	0	0	0	4	0	0	T2N0M0	-	II	2	Malignant	Mpr060006
A7	7	1	72	M	Prostate	Adenocarcinoma	0	0	0	4	1	4	T2N0M0	1	I	1	Malignant	Mpr040048
A8	8	1	76	M	Prostate	Adenocarcinoma	0	0	0	4	1	4	T2N0M0	2	II	2	Malignant	Mpr040106
A9	9	1	71	M	Prostate	Adenocarcinoma	0	0	0	4	1	4	T2N0M0	1	II	2	Malignant	Mpr070077
A10	10	1	71	M	Prostate	Adenocarcinoma	0	0	0	4	1	4	T2N0M0	2	II	2	Malignant	Mpr080033
B1	11	1	71	M	Prostate	Adenocarcinoma	0	0	0	0	0	0	T1N0M0	2	I	1	Malignant	Mpr080053
B2	12	0	65	M	Prostate	Adenocarcinoma	0	0	0	4	1	4	T2N0M0	2	II	2	Malignant	Mpr030031
B3	13	1	75	M	Prostate	Adenocarcinoma	1	1	0	4	1	4	T2N1M1C	1	IV	4	Malignant	Mpr030402
B4	14	0	60	M	Prostate	Adenocarcinoma	0	0	0	4	1	4	T2N0M0	2	II	2	Malignant	Mpr020111
B5	15	0	67	M	Prostate	Adenocarcinoma	0	1	0	4	2	8	T2N0M1	2	IV	4	Malignant	Mpr030220
B6	16	0	60	M	Prostate	Adenocarcinoma	1	0	1	4	1	4	T3N1M0	2	IV	4	Malignant	Mpr080011
B7	17	1	71	M	Prostate	Adenocarcinoma	0	0	0	0	0	0	T2N0M0	2	IIA	2	Malignant	Mpr130007
B8	18	1	75	M	Prostate	Adenocarcinoma	1	1	1	4	3	12	T4N1M1	2	IV	4	Malignant	Mpr030207
B9	19	1	71	M	Prostate	Adenocarcinoma	0	0	0	4	1	4	T1N0M0	2	I	1	Malignant	Mpr130042
B10	20	0	66	M	Prostate	Adenocarcinoma	0	0	0	4	0	0	T2N0M0	2	IIA	2	Malignant	Mpr130012
C1	21	0	64	M	Prostate	Adenocarcinoma	0	1	1	4	2	8	T3N0M1	2	IV	4	Malignant	Mpr030359
C2	22	1	71	M	Prostate	Adenocarcinoma	0	0	0	4	1	4	T2N0M0	2	II	2	Malignant	Mpr050123
C3	23	0	64	M	Prostate	Adenocarcinoma	0	0	0	4	1	4	T2N0M0	3	II	2	Malignant	Mpr010054
C4	24	1	73	M	Prostate	Adenocarcinoma	0	0	0	4	1	4	T2N0M0	3	II	2	Malignant	Mpr020053
C5	25	0	56	M	Prostate	Adenocarcinoma	0	0	0	0	0	0	T2N0M0	2	II	2	Malignant	Mpr040206
C7	27	0	70	M	Prostate	Adenocarcinoma	1	1	0	4	1	4	T2N1M1C	2	IV	4	Malignant	Mpr020345
C8	28	1	82	M	Prostate	Adenocarcinoma	1	0	1	4	3	12	T3N1M0	3	IV	4	Malignant	Mpr100019
C9	29	0	64	M	Prostate	Adenocarcinoma	0	0	0	0	0	0	T2N0M0	3	IB	1	Malignant	Mpr070118
C10	30	0	61	M	Prostate	Adenocarcinoma	0	0	0	4	1	4	T2N0M0	2–3	II	2	Malignant	Mpr070019
D1	31	0	69	M	Prostate	Adenocarcinoma	0	0	1	4	3	12	T3N0M0	2–3	III	3	Malignant	Mpr030166
D2	32	1	73	M	Prostate	Adenocarcinoma	1	1	1	4	2	8	T3N1M1C	3	IV	4	Malignant	Mpr030374
D3	33	0	65	M	Prostate	Adenocarcinoma	0	0	0	4	1	4	T2N0M0	3	II	2	Malignant	Mpr040034
D4	34	1	74	M	Prostate	Adenocarcinoma	0	0	0	4	1	4	T2N0M0	-	IIA	2	Malignant	Mpr130029
D5	35	0	20	M	Prostate	Adenocarcinoma	0	0	1	4	2	8	T3NOMO	3	III	3	Malignant	Mpr060171
D6	36	0	70	M	Prostate	Adenocarcinoma						0	-	3	-		Malignant	Mpr060100
D7	37	1	73	M	Prostate	Adenocarcinoma	0	0	0	4	1	4	T2N0M0	3	II	2	Malignant	Mpr050122
D8	38	1	73	M	Prostate	Adenocarcinoma	1	0	1	4	1	4	T3N1M0	3	IV	4	Malignant	Mpr030433
D9	39	1	72	M	Prostate	Adenocarcinoma	0	0	1	4	1	4	T3N0M0	3	III	3	Malignant	Mpr050093
D10	40	0	64	M	Prostate	Adenocarcinoma	0	0	1	4	2	8	T3N0M0	2–3	III	3	Malignant	Mpr020003
E1	41	1	74	M	Prostate	Adenocarcinoma	0	0	0	4	1	4	T2N0M0	3	II	2	Malignant	Mpr040041
E2	42	1	82	M	Prostate	Adenocarcinoma	0	0	0	4	1	4	T2N0M0	3	II	2	Malignant	Mpr030147
E3	43	0	68	M	Prostate	Adenocarcinoma	0	0	0	4	0	0	T2N0M0	3	II	2	Malignant	Mpr130039
E4	44	0	66	M	Prostate	Adenocarcinoma	0	0	1	4	1	4	T3AN0M0	3	III	3	Malignant	Mpr060047
E5	45	0	57	M	Prostate	Adenocarcinoma	0	0	0	4	1	4	T2N0M0	3	II	2	Malignant	Mpr130032
E6	46	1	82	M	Prostate	Adenocarcinoma	1	0	1	3	3	9	T3N1M0	3	IV	4	Malignant	Mpr100019
E7	47	0	64	M	Prostate	Adenocarcinoma	0	0	0	4	1	4	T2N0M0	3	II	2	Malignant	Mpr130033
E8	48	1	76	M	Prostate	Adenocarcinoma	0	0	1	4	0	0	T3N0M0	3	III	3	Malignant	Mpr020387
E9	49	1	78	M	Prostate	Adenocarcinoma	1	1	1	4	2	8	T4N1M1	3	IV	4	Malignant	Mpr030228
E10	50	1	75	M	Prostate	Adenocarcinoma	0	0	0	4	1	4	T2N0M0	2	IIA	2	Malignant	Mpr040053
F1	51	0	62	M	Prostate	Adenocarcinoma	0	0	1	4	1	4	T3N0M0	3	III	3	Malignant	Mpr040089
F2	52	0	70	M	Prostate	Adenocarcinoma	0	0	0	4	1	4	T2N0M0	-	II	2	Malignant	Mpr020038
F3	53	0	70	M	Prostate	Adenocarcinoma	0	0	1	4	1	4	T3N0M0	3	IIB	2	Malignant	Mpr020106
F4	54	0	64	M	Prostate	Adenocarcinoma	0	0	0	4	0	0	T2AN0M0	3	II	2	Malignant	Mpr040171
F5	55	0	62	M	Prostate	Adenocarcinoma	0	0	0	4	0	0	T2N0M0	3	II	2	Malignant	Mpr040163
F6	56	1	77	M	Prostate	Adenocarcinoma	0	0	0	4	0	0	T2N0M0	3	II G4	2	Malignant	Mpr040215
F7	57	0	60	M	Prostate	Adenocarcinoma	1	0	1	4	2	8	T3N1M0	3	IV	4	Malignant	Mpr030236
F9	59	0	56	M	Prostate	Adenocarcinoma	0	0	1	0	0	0	T3N0M0	3	III	3	Malignant	Mpr130006
F10	60	0	67	M	Prostate	Adenocarcinoma	0	0	0	4	1	4	T2N0M0	3	II	2	Malignant	Mpr040165
G1	61	1	87	M	Prostate	Adenocarcinoma	0	0	0	4	1	4	T2N0M0	3	II	2	Malignant	Mpr020242
G2	62	1	81	M	Prostate	Adenocarcinoma	0	0	1	4	1	4	T3N0M0	3	III	3	Malignant	Mpr020317
G3	63	1	73	M	Prostate	Adenocarcinoma	0	0	0	4	1	4	T2BN0M0	3	IIA	2	Malignant	Mpr130034
G4	64	0	69	M	Prostate	Adenocarcinoma	0	0	0	4	0	0	T2N0M0	3	II	2	Malignant	Mpr030352
G5	65	0	62	M	Prostate	Adenocarcinoma	0	0	0	4	0	0	T2N0M0	3	II	2	Malignant	Mpr050083
G6	66	0	64	M	Prostate	Adenocarcinoma	0	0	0	0	0	0	T2N0M0	3	II	2	Malignant	Mpr060077
G7	67	1	82	M	Prostate	Adenocarcinoma	0	0	1	4	1	4	T3N0M0	3	III	3	Malignant	Mpr040075
G8	68	1	80	M	Prostate	Adenocarcinoma	1	1	1	4	2	8	T4N1M1C	3	IV	4	Malignant	Mpr020382
G9	69	0	63	M	Prostate	Adenocarcinoma	0	0	0	4	1	4	T2N0M0	3	II	2	Malignant	Mpr030347
G10	70	1	73	M	Prostate	Adenocarcinoma	0	0	1	4	2	8	T4N0M0	3	IV	4	Malignant	Mpr030188
H1	71	0	60	M	Prostate	Adenocarcinoma	0	0	0	4	1	4	T2N0M0	3	II	2	Malignant	Mpr020323
H4	74		27	M	Prostate	Adjacent normal prostate	NAT	NAT	NAT			0	-	-	-		NAT	Mpr040012
H5	75		35	M	Prostate	Adjacent normal prostate	NAT	NAT	NAT			0	-	-	-		NAT	Mpr070058
H6	76		21	M	Prostate	Adjacent normal prostate	NAT	NAT	NAT			0	-	-	-		NAT	Mpr040014
H7	77		45	M	Prostate	Prostate tissue	Normal	Normal	Normal			0	-	-	-		Normal	Mpr07N030
H8	78		37	M	Prostate	Prostate tissue	Normal	Normal	Normal			0	-	-	-		Normal	Mpr07N028
H9	79		33	M	Prostate	Prostate tissue	Normal	Normal	Normal			0	-	-	-		Normal	Mpr07N026
H10	80		43	M	Prostate	Prostate tissue	Normal	Normal	Normal			0	-	-	-		Normal	Mpr06N019

(5) Supplementary Table 2

DU145- ID	MS2 name	MS2 score	type	mz	rt	MEAN NC	MEAN SH	VIP	P-VALUE	Q-VALUE	FOLD CHANGE	LOG_FOLDCHANGE
5241	1-Myristoyl-sn-glycero-3-phosphocholine	0.9491	MS2 forward	468.3097873	181.641	0.068968236	0.113517602	1.54210749	1.14085E-06	4.67872E-06	1.645940347	0.71891205
296	Creatinine	0.9972	MS2 forward	114.066953	160.505	0.026430593	0.045546402	1.432148478	4.11946E-05	6.46202E-05	1.723245581	0.785128316
516	Methyl acetoacetate	0.9958	MS2 reverse	134.0817286	329.328	0.01158646	0.018453727	1.45324927	1.92524E-05	3.57749E-05	1.592697637	0.671472407
860	L-Carnitine	0.9481	MS2 forward	162.1135707	357.413	0.252475197	0.36006097	1.215944268	0.004806181	0.003110176	1.426124132	0.512099561
1946	Lys-Val	0.9925	MS2 reverse	246.1821592	525.181	0.016809016	0.024942622	1.320800858	0.000681673	0.000634278	1.483883473	0.569377804
315	L-Proline	0.9999	MS2 forward	116.0716176	294.863	0.351689836	0.718513607	1.532948235	3.32496E-06	1.01032E-05	2.043032052	1.030711838
5806	1-O-Octadecyl-sn-glyceryl-3-phosphorylcholine	0.9861	MS2 forward	510.3909364	177.954	0.040523993	0.029504673	1.046237882	0.021175804	0.010144726	0.728079124	-0.45783285
428	Taurine	0.9964	MS2 forward	126.0229543	277.711	0.186688682	0.413739794	1.51592918	2.32429E-06	7.75866E-06	2.216201812	1.148089262
287	Uracil	0.9464	MS2 forward	113.035166	89.4825	0.016498488	0.021035203	1.096009936	0.026845176	0.012363597	1.274977647	0.350471954
3002	Tyr-Met	0.9703	MS2 forward	312.1118534	163.65	0.030122583	0.022746909	1.453447627	0.000680762	0.000633565	0.755144709	-0.405174958
5423	1-Stearoyl-2-hydroxy-sn-glycero-3-phosphoethanolamine	0.6549	MS2 forward	482.3274229	179.784	0.101271127	0.153188719	1.482783748	8.2559E-06	1.98119E-05	1.512659364	0.597087143
2035	Deoxyadenosine	0.9967	MS2 forward	252.1103974	137.571	0.047735591	0.080148641	1.441449248	5.79021E-05	8.43451E-05	1.679012234	0.747612743
845	Cyclohexylamine	0.736	MS2 forward	160.1340962	366.77	0.134568657	0.248882508	1.476320113	1.68106E-05	3.26009E-05	1.849483487	0.88712242
552	Hypoxanthine	0.9851	MS2 forward	137.0468347	161.13	0.856308413	0.305811013	1.573326569	2.98852E-08	2.5103E-07	0.357127185	-1.485490135
1532	N-Acetylserotonin	0.9363	MS2 forward	219.1138801	42.048	0.052150172	0.080338575	1.144200633	0.006698838	0.004055303	1.540523678	0.623420857
5053	1-Palmitoyl-2-hydroxy-sn-glycero-3-phosphoethanolamine	0.9854	MS2 forward	454.2934799	183.947	0.04384008	0.060804718	1.422475659	7.24642E-05	0.000101115	1.386966398	0.471932836
1543	Pantothenate	0.6664	MS2 forward	220.1192291	253.75	0.010150136	0.007369832	1.325302852	0.000903021	0.000802568	0.726082122	-0.461795365
669	(3-Carboxypropyl)trimethylammonium cation	0.9407	MS2 forward	146.1187644	358.746	0.247834251	0.473767062	1.502070857	1.26983E-05	2.69911E-05	1.911628683	0.93480232
229	Picolinic acid	0.6392	MS2 forward	106.0293815	61.8615	0.05022723	0.004686963	1.581571957	3.4249E-05	5.6471E-05	0.093315181	-3.421744378
717	Triethanolamine	0.9353	MS2 forward	150.1131989	281.019	0.019621289	0.013134871	1.558134163	1.02155E-06	4.31364E-06	0.669419409	-0.579017715
2211	Creatine	0.9929	MS2 reverse	263.1471411	329.269	0.00327034	0.008574147	1.401040407	4.71717E-05	7.10241E-05	2.621790668	1.390552501
1378	Acetylcarnitine	0.9997	MS2 forward	204.1244643	288.22	0.804654796	2.91169269	1.517057572	0.000190931	0.00021074	3.618561283	1.855416205
365	Tyramine	0.6795	MS2 forward	120.0813508	245.086	0.041628443	0.060250713	1.381416568	0.000343347	0.000350132	1.447344874	0.533408729
9876	N-Docosanoyl-4-sphingenyl-1-O-phosphorylcholine	0.9672	MS2 forward	787.6675736	157.826	0.054259561	0.004626597	1.427348229	0.005005709	0.003210103	0.085267871	-3.55185396
385	Nicotinamide	0.9903	MS2 forward	123.056318	61.842	21.57897696	2.535547477	1.593663055	1.64989E-05	3.22102E-05	0.11750082	-3.089257276
**5760**	**Adenosine 5'-triphosphate (ATP)**	**0.9975**	**MS2 forward**	**508.0032413**	**480.582**	**0.053789625**	**0.038646732**	**1.288705043**	**0.002851534**	**0.002040259**	**0.718479302**	**-0.476981499**
2302	Adenosine	1	MS2 forward	268.1062302	162.349	14.26456311	10.63037783	1.178492052	0.013617389	0.007149147	0.745229822	-0.424242687
537	Adenine	0.9993	MS2 forward	136.0634393	151.338	5.779814743	1.051215114	1.568461243	0.00015769	0.000183008	0.181876956	-2.458965328
2315	Inosine	0.9998	MS2 forward	269.0892839	206.488	0.115777735	0.029892546	1.570298657	3.29039E-05	5.47955E-05	0.258189071	-1.95350016
1944	2-Methylbutyroylcarnitine	0.9947	MS2 forward	246.1721331	223.692	0.368477747	2.627169961	1.586641894	5.28797E-06	1.39551E-05	7.129792727	2.833860136
2412	1,2,3-Benzenetriol	0.8925	MS2 reverse	275.0539118	384.625	0.007756626	0.002965457	1.528027054	0.000734162	0.000674848	0.382312798	-1.3871746
1915	Uridine	0.9913	MS2 forward	245.077937	154.749	0.010133541	0.006137275	1.318885233	0.000752486	0.00068878	0.605639723	-0.723468263
746	2-Hydroxyadenine	0.868	MS2 forward	152.0574864	214.36	0.14460858	0.054253653	1.530458694	9.18774E-05	0.000121392	0.375175895	-1.414360958
129	L-Alanine	0.9999	MS2 forward	90.05579365	329.328	0.147753573	0.237192951	1.446721546	1.78035E-05	3.3811E-05	1.605328022	0.682868118
4711	Stearoylcarnitine	0.9978	MS2 forward	428.3735888	152.81	0.326475632	1.404915078	1.584036808	4.0803E-11	9.63406E-10	4.303277002	2.10543571
4338	L-Palmitoylcarnitine	0.9924	MS2 forward	400.3440676	156.009	0.767583325	4.033443981	1.588242307	3.68793E-12	2.49803E-10	5.254731115	2.393616943
5706	1-Eicosatrienoyl-sn-glycero-3-phosphoethanolamine	0.954	MS2 reverse	504.306641	179.46	0.033961813	0.039137701	1.305526992	0.001233583	0.001030965	1.152403183	0.204645551
2914	Cytidine 2',3'-cyclic phosphate	0.9985	MS2 forward	306.0496902	301.109	0.010396838	0.007483363	1.165704631	0.012987244	0.006882329	0.719772985	-0.474386139
916	L-Phenylalanine	0.9599	MS2 forward	166.0868713	245.32	0.025514705	0.035401957	1.308894926	0.00107916	0.000927596	1.387511883	0.472500127

(6) Supplementary Table 3

PC3-ID	MS2 name	MS2 score	type	mz	rt	MEAN NC	MEAN SH	VIP	P-VALUE	Q-VALUE	FOLD CHANGE	LOG_FOLDCHANGE
5494	1-Myristoyl-sn-glycero-3-phosphocholine	0.9325	MS2 forward	468.3083391	181.812	0.051046986	0.066798835	1.522114272	0.000397188	0.001443145	0.764189763	-0.387997163
299	Creatinine	0.9987	MS2 forward	114.0648888	160.394	0.050676825	0.065915466	1.205107778	0.013229476	0.021859852	0.768815395	-0.37929087
531	Methyl acetoacetate	0.9958	MS2 reverse	134.0794154	49.599	0.017904285	0.026126949	1.094131948	0.026619252	0.036683579	0.685280336	-0.545233804
895	L-Carnitine	0.9813	MS2 forward	162.1114862	336.82	0.376115916	0.623866035	1.521112692	0.000143403	0.000660687	0.602879295	-0.730058911
1959	Lys-Val	0.9925	MS2 reverse	246.1801166	528.129	0.030403031	0.037540245	1.063641588	0.036675496	0.045339083	0.809878316	-0.304222935
320	L-Proline	0.9999	MS2 forward	116.0698684	294.923	0.260733768	0.456145467	1.594047638	7.71951E-05	0.000446021	0.571602234	-0.806916539
6145	1-O-Octadecyl-sn-glyceryl-3-phosphorylcholine	0.8576	MS2 forward	510.390612	178.4305	0.048494489	0.015407819	1.496443835	0.001965556	0.00484653	3.147394739	1.654158129
437	Taurine	0.9972	MS2 forward	126.0208209	278.174	0.055159251	0.076182696	1.32047643	0.003554675	0.007403907	0.724039099	-0.465860488
288	Uracil	0.9824	MS2 forward	113.0332983	155.063	0.180321127	0.274338426	1.438412016	0.000673411	0.002177922	0.65729446	-0.60538827
3064	Tyr-Met	0.9712	MS2 forward	312.1100801	162.255	0.061431098	0.080911317	1.369783457	0.002646882	0.006000968	0.759239871	-0.397372338
5687	1-Stearoyl-2-hydroxy-sn-glycero-3-phosphoethanolamine	0.6575	MS2 forward	482.3257604	179.05	0.034055956	0.105035231	1.736079849	1.99857E-08	1.79263E-06	0.324233647	-1.624894284
2050	Deoxyadenosine	0.9995	MS2 forward	252.1082142	137.501	0.062826506	0.211394248	1.594028763	1.82117E-07	5.2982E-06	0.297200641	-1.750490866
879	Cyclohexylamine	0.7269	MS2 forward	160.131509	366.971	0.026902675	0.032241402	1.052495842	0.041941759	0.050180614	0.834413922	-0.261164866
568	Hypoxanthine	0.9909	MS2 forward	137.0446234	160.392	0.342590181	1.156474829	1.737664854	1.91795E-08	1.7569E-06	0.296236609	-1.755178157
1573	N-Acetylserotonin	0.9481	MS2 forward	219.1118279	40.851	0.053406017	0.080104907	1.442729285	0.001770455	0.004477754	0.66670094	-0.584888333
5287	1-Palmitoyl-2-hydroxy-sn-glycero-3-phosphoethanolamine	0.9926	MS2 forward	454.2925385	183.823	0.041901617	0.053356606	1.570140097	0.000240187	0.000962276	0.785312653	-0.348660953
1588	Pantothenate	0.9434	MS2 forward	220.1168798	254.138	0.034304455	0.055018502	1.456015161	0.000962474	0.002837463	0.623507623	-0.681520895
690	(3-Carboxypropyl)trimethylammonium cation	0.8707	MS2 forward	146.1162108	359.591	0.045306047	0.052814246	1.093117653	0.02826986	0.038232243	0.857837616	-0.221223515
410	Picolinic acid	0.9972	MS2 reverse	124.0376372	201.552	0.002220327	0.00486091	1.607155176	5.90545E-05	0.000371975	0.456771815	-1.130454464
765	Triethanolamine	0.8838	MS2 forward	150.1108306	283.742	0.00836664	0.010071315	1.004523146	0.042112434	0.050335242	0.830739518	-0.267531911
2875	Creatine	0.9948	MS2 reverse	301.1019067	329.773	0.002746639	0.00383197	1.152142637	0.031101452	0.040759099	0.716769286	-0.480419277
1407	Acetylcarnitine	0.9997	MS2 forward	204.1226211	289.283	2.601275519	3.492838604	1.123109978	0.02641813	0.036494509	0.74474541	-0.425180768
369	Tyramine	0.9125	MS2 forward	120.0794312	239.513	0.046279365	0.080574026	1.65401774	1.11222E-05	0.000107925	0.574370758	-0.799945792
11867	N-Docosanoyl-4-sphingenyl-1-O-phosphorylcholine	0.9979	MS2 reverse	901.5572369	113.377	0.016229189	0.021990181	1.367313907	0.001557422	0.004052789	0.7380198	-0.438268572
395	Nicotinamide	0.999	MS2 forward	123.0540603	145.178	0.266756219	0.473401217	1.533981268	0.000537019	0.001821401	0.563488663	-0.82754151
**6099**	**Adenosine 5'-triphosphate (ATP)**	**0.9942**	**MS2 forward**	**508.0028273**	**459.231**	**0.093786737**	**0.046590666**	**1.543146052**	**0.000515779**	**0.00176722**	**2.012994133**	**1.009342968**
7276	Adenosine	1	MS2 reverse	573.1449632	347.978	0.004798604	0.008632445	1.602802496	1.13819E-05	0.000109495	0.555879974	-0.847154687
549	Adenine	0.9997	MS2 forward	136.0614794	151.182	3.544003932	8.871405509	1.711573093	6.4089E-08	3.39097E-06	0.399486184	-1.323782486
2356	Inosine	0.9999	MS2 forward	269.0874033	205.927	0.048983303	0.196437822	1.726958139	1.07533E-07	4.37208E-06	0.2493578	-2.003710763
1958	2-Methylbutyroylcarnitine	0.9925	MS2 forward	246.1688287	224.866	0.03087749	0.040307922	1.171685713	0.014558001	0.023483722	0.766040226	-0.384507942
2449	1,2,3-Benzenetriol	0.9085	MS2 reverse	275.051415	382.98	0.003446621	0.002081946	1.597811494	0.003104107	0.006676787	1.65548092	0.727250383
1933	Uridine	0.9972	MS2 forward	245.0757848	155.045	0.029190351	0.044552607	1.530018421	0.000122899	0.000591149	0.655188407	-0.610018265
1039	2-Hydroxyadenine	0.9673	MS2 forward	174.0368637	247.3885	0.002073995	0.005003285	1.687230991	0.000386892	0.001413152	0.414526759	-1.270462858
118	L-Alanine	0.998	MS2 forward	90.05411368	329.783	0.229790623	0.253552673	1.125160509	0.035611011	0.044475424	0.906283577	-0.141965553
4887	Stearoylcarnitine	0.995	MS2 forward	428.3731202	153.07	0.358722784	0.240405503	1.628751136	8.41251E-06	8.98291E-05	1.492157125	0.57739946
4444	L-Palmitoylcarnitine	0.993	MS2 forward	400.3428183	156.421	2.20768593	0.703972216	1.745655367	3.24124E-10	8.57477E-08	3.13604128	1.64894455
6042	1-Eicosatrienoyl-sn-glycero-3-phosphoethanolamine	0.9738	MS2 reverse	504.3066808	179.833	0.027562556	0.035258665	1.593737269	0.000160878	0.000717608	0.781724322	-0.35526817
2958	Cytidine 2',3'-cyclic phosphate	0.9992	MS2 forward	306.0492931	396.48	0.003361139	0.005048633	1.316962706	0.011994412	0.020290699	0.665752314	-0.586942558
939	L-Phenylalanine	0.9964	MS2 forward	166.0848735	239.617	0.027264047	0.040976299	1.620609336	4.83898E-05	0.000329564	0.665361394	-0.587789935
